# Stock returns predictability and the adaptive market hypothesis in emerging markets: evidence from India

**DOI:** 10.1186/2193-1801-3-428

**Published:** 2014-08-12

**Authors:** Gourishankar S Hiremath, Jyoti Kumari

**Affiliations:** Department of Humanities and Social Sciences, Indian Institute of Technology Kharagpur, Kharagpur, 721302 India

**Keywords:** Adaptive market hypothesis, Market efficiency, Random walk, Autocorrelation, Nonlinearity, Predictability, Financial crisis, Evolving efficiency, Emerging markets

## Abstract

**Abstract:**

This study addresses the question of whether the adaptive market hypothesis provides a better description of the behaviour of emerging stock market like India. We employed linear and nonlinear methods to evaluate the hypothesis empirically. The linear tests show a cyclical pattern in linear dependence suggesting that the Indian stock market switched between periods of efficiency and inefficiency. In contrast, the results from nonlinear tests reveal a strong evidence of nonlinearity in returns throughout the sample period with a sign of tapering magnitude of nonlinear dependence in the recent period. The findings suggest that Indian stock market is moving towards efficiency. The results provide additional insights on association between financial crises, foreign portfolio investments and inefficiency.

**JEL codes:**

G14; G12; C12

## 1 Background

There is no theory that has attracted volumes of research like efficient market hypothesis (EMH) over four decades. It is the well-known, yet highly controversial theory of Neoclassical School of Finance, which has influenced modern finance in both theory and practice. A market is efficient when prices always ‘fully reflect’ available information (Fama 1970)^a^. In such an efficient market, when new information arrives, security prices quickly respond and incorporate all information at any point of time, and reach a new equilibrium. Moreover, collection of information is costly and there will be no extra returns on such actions in an informationally efficient market. The fundamental or technical analysis cannot outperform a simple strategy of buying and holding diversified securities. In other words, the EMH rules out any active portfolio management^b^.

Despite a large body of research on EMH both from developed and developing markets, the consensus on this issue that whether markets are efficient or not, thus continues to be elusive. In recent years, although there is striking evidence that stock returns do not follow random walk and possess some components of predictability, there is a lack of strong alternative theoretical explanations to EMH. Nevertheless, using an evolutionary approach to economic interaction, Lo (2004) has proposed the adaptive market hypothesis (AMH) which can coexist with the EMH in an intellectually consistent manner. The emerging and developing markets have more tendencies to reject EMH because of several market frictions. Unlike EMH that assumes a frictionless market, AMH accommodates market frictions and asserts that markets evolve over a period. In light of this, the present article aims to determine whether AMH provides a better description of the Indian stock market, one of the emerging markets. To the best of our knowledge, there are no studies of this kind in India.

Lo (2004) offers an alternative market theory to EMH from a behavioural perspective, according to which, markets are adaptable and switch between efficiency and inefficiency at different points of time. Lo (2004) applies the evolutionary approach of biology to economic interactions and explains the adaptive nature of the agents and consequently how market becomes adaptive. According to Lo (2005), “degree of market efficiency is related to environmental factors characterizing market ecology such as the number of competitors, the magnitude of profit opportunities available, and the adaptability of the market participants. In contrast to EMH, which assumes a frictionless market, AMH asserts that the laws of natural selection or “survival of the richest” determines the evolution of markets and institutions in real world markets, which have frictions.

Unlike investors in efficient markets, investors do make mistakes and learn to adapt their behaviour accordingly in the framework of AMH. The AMH has a number of practical implications. First, the risk-reward relationship changes over time because of the preferences of the populations in the market. Second, the movement of past prices influences the current preferences because of the forces of natural selection and thus AMH contrasts the weak form of efficiency where history of prices is of no use. Third, in adaptive market, arbitrage opportunities do exist from time to time. From an evolutionary perspective, the profit opportunities are being constantly created and disappear. This calls for investment strategies according to the market environment. In other words, AMH implies “complex market dynamics” which necessitates the active portfolio management. Fourth, innovation is a key to survival and AMH suggest adapting to changing market conditions to ensure a consistent level of expected returns. Finally, market efficiency is not an all or none condition but a characteristic that varies continuously over time and across markets^c^. Hence, a financial market may witness the periods of efficiency and inefficiency.

The AMH though still in its infancy is attracting attention from researchers. Ito and Sugiyama (2009) find time varying market inefficiency in the US. Charles et al. (2012) holds AMH true in case foreign exchange rates of developing countries in which they find episodes of return predictability depending on market conditions. Kim *et al*. (2011) tests whether the US stock market evolved over time in the US. They find market conditions as the driving factors of predictability and market is more efficient after 1980s than the previous periods. Exploring the relative efficiency, Noda (2012) concludes that TOPIX support AMH while TSE2 rejects AMH in case of Japan. Alvarez-Ramirez *et al.* (2012) provide evidence in favour of AMH and find the US market more efficient during 1973 to 2003. Urquhart and Hudson (2013) document mixed results for the US, the UK and Japan and conclude that the AMH provides a convincing description of these markets.

Given the importance of AMH, a fresh study of efficiency of Indian stock market is required for the following reasons. First, a limited number of studies empirically tested EMH in context of India but the findings are mixed (E.g. Rao and Mukherjee 1971; Sharma and Kennedy 1977; Barua 1981; Amanulla and Kamaiah 1998; Poshakwale 2002, Hiremath and Kamaiah 2010, 2012 among others). There are no studies on Indian stock market, which investigated adaptive behaviour of stock markets. Second, the economic reforms in India were introduced in early 1990s to infuse energy and vibrancy to the process of economic growth. In addition, capital market plans with setting up of National Stock Exchange (NSE) and changes in the market microstructure and trading practices from 1994 sought a transparent, fair and efficient market. As a result, India’s financial system grew by leaps and bounds in post liberalization era. As per the S & P Fact book (Standard and Poor’s 2012), Indian stock market now has the largest number of listed companies on its exchanges. The growing percentage of market capitalization to the GDP and the increasing integration of the Indian market with the global economy indicate the phenomenal growth of the Indian equity market and its growing importance in the economy. Therefore, it is reasonable to expect that emerging markets like India exhibit different characteristics, which distinguish it from developed stock markets and such features influence the nature of market efficiency. Third, the capital market of India emerged as one of the important destinations for investment. The keen interest of foreign institutional investors (FIIs) in Indian stock market for portfolio diversification and higher expected returns is evident from surging foreign investment into Indian capital market. The yield sensitive portfolio investments positively offer liquidity to local markets and sometime trigger panic in the market by reversing the investments. It is logical to expect influence of FIIs on efficiency. Finally, financial crises, both of domestic and foreign origin may affect efficiency of local financial markets.

In this light, departing from the previous studies on efficiency of Indian stock market, we make the following contributions. To the best of our knowledge, this is the first comprehensive work on Indian stock market, which examines the AMH. Thus, the present article complements literature on AMH and extends existing work that has examined efficiency of Indian stock market. Essentially, the available studies refer to the 1980s and early 1990s and hence could not capture the changes in the nature of stock market efficiency in the post policy reforms era. The present study covers the period (1991 to 2013) of such changes is in order. Further, the present study employs methods and techniques, which are first of their kind in the Indian context. Finally, the issue of nonlinearity in stock returns is addressed in this paper seldom received due attention in India. The remainder of the article is structured as follows. Section 2 describes data and econometric methods implemented for estimations. Section 3 discusses the main results and evaluates the relevance of AMH for India. Section 4 summarizes and concludes.

## 2 Methods

For empirical testing, we use daily values of Sensex and Nifty, the major indices traded in India and together constitute 99.9 percent of total market capitalization. The Sensex data is from January 1991 to March 2013 while Nifty data spans from January 1994 to March 2013. To capture changing efficiency or evolving nature of the market, we divide the whole sample into two yearly subsamples^d^. The present study employs both linear and nonlinear tests for empirical testing of AMH. The sample characteristics and a set of the tests make the results of the present study robust and reduce the risk of overemphasizing the generality of the findings. The following subsections offer a brief description of these tests.

### 2.1 Linear Tests

#### 2.1.1 Autocorrelation Test

Autocorrelation estimates are used to test the hypothesis that the process generating the observed return is a series of independent and identical distribution (*iid*) of random variables. It helps to evaluate whether successive values of serial correlation are different from zero. To test the joint hypothesis that all autocorrelation coefficients *ρ*_*k*_ are simultaneously equal to zero, we use Ljung and Box’s (1978) portmanteau Q statistic. The test statistic is
1

where *n* is the number of observations, *m* lag length. The test follows a chi-square (χ^2^) distribution.

#### 2.1.2 Runs Test

Runs test is one of the prominent nonparametric tests of the random walk hypothesis (RWH). A run is defined as the sequence of consecutive changes in the return series. If the sequence is positive (negative), it is a positive (negative) run and if there are no changes in the series, then a run is zero. The expected runs are the change in returns required, if a random process generates the data. If the actual runs are close to the expected number of runs, it indicates that the returns are generated by a random process. The expected number of runs (ER) is computed as
2

where X is the total number of runs, c_*i*_ is the number of returns changes of each category of sign (*i =* 1, 2, 3). The ER in Equation (2) has an approximate normal distribution for large X. Hence, to test the null hypothesis, we use standard Z statistic.

#### 2.1.3 Variance Ratio Test

Lo and MacKinlay (1988) proposed the variance ratio test which is capable of distinguishing between several interesting alternative stochastic processes. Under RWH for stock returns r_*t*_, the variance of r_*t*_ + r_*t*-1_ are required to be twice the variance of r_*t*_. Let the ratio of the variance of two period returns, r_*t*_(2) ≡ r_*t*_ - r_*t* - 1_, to twice the variance of a one-period return r_*t*_. Then variance ratio VR (2) is
3

where *ρ* (1) is the first order autocorrelation coefficient of returns {r_*t*_}. RWH which requires zero autocorrelations holds true when VR (2) =1. The VR (2) can be extended to any number of period returns, *q*. Lo and MacKinlay (1988) showed that the *q* period variance ratio satisfies the following relation:
4

where r_*t*_(*k*) ≡ r_*t*_ + r_*t* - 1_ + … + r_*t* - *k* + 1_ and *ρ* (*k*) is the *k*^th^ order autocorrelation coefficient of {r_*t*_} Equation (4) shows that at all *q,* VR (*q*) = 1. For random walk to hold, variance ratio is expected to be equal to unity. The test is based on standard asymptotic approximations. Lo-MacKinlay proposed Z (*q*) standard normal test statistic under the null hypothesis of homoscedastic increments and VR (*q*) =1. The rejection of RWH because of heteroscedasticity, a common feature of financial returns is not useful for any practical purpose. Hence, Lo-MacKinlay constructed a heteroscedastic robust test statistic Z* (*q*) which can be defined as
5

which follows a standard normal distribution asymptotically. Thus, according to variance ratio test, the returns process is a random walk when the variance ratio at a holding period *q* is a unity. The variance ratio less than unity imply negative autocorrelation and greater than one indicates positive autocorrelation.

#### 2.1.4 Multiple Variance Ratio Test

The variance ratio of Lo and MacKinlay (1988) tests whether the variance ratio is equal to one for a particular holding period, whereas the RWH requires that variance ratios for all holding periods should be equal to one and the test should be conducted jointly over a number of holding periods. The sequential procedure of this test leads to size distortions and the test ignores the joint nature of random walk. To overcome this problem, Chow and Denning (1993) proposed multiple variance ratio test wherein a set of multiple variance ratios over a number of holding periods are tested to determine whether the multiple variance ratios (over a number of holing periods) are jointly equal to one. In Lo-MacKinlay test, under the null, VR (*q*) = 1, but in multiple variance ratio test, *M*_*r*_ = (*q*_*i*_) = *VR* (*q*) – 1 = 0 which is generalized to a set of *m* variance ratio tests as
6

Under RWH, multiple and alternative hypotheses are as follows
7a7b

The null of random walk is rejected when any one or more of H_0*i*_ is rejected. The heteroscedastic test statistic in Chow-Denning is:
8

where Z*(*q*_*i*_) is defined as in Equation (5). Chow-Denning test follows studentized maximum modulus, SMM(α, *m*, T), distribution with *m* parameters and T degrees of freedom. The RWH is rejected, if the value of the standardized test statistic CD. is greater than the SMM critical values at the chosen significance level.

### 2.2 Nonlinear tests

To test the presence of nonlinear dependence, we have carried out a set of nonlinear tests to avoid sensitivity of empirical results to the test employed. Before performing these tests, the linear dependence is removed from the data through fitting AR (*p*). The optimal lag is selected so that no Ljung-Box (LB) Q statistic for residuals extracted from AR (*p*) model is significant at 1 per cent level. Besides, we corrected the financial returns for heteroscedasticity. Therefore, rejection of null for residuals implies presence of nonlinear dependence in returns and market inefficiency.

#### 2.2.1 McLeod-Li Test

McLeod and Li’s (1983) portmanteau test of nonlinearity seeks to discover whether the squared autocorrelation function of returns is non-zero. The test statistic is
9

where  is the autocorrelation of the squared residuals and  is obtained after fitting appropriate AR (*p*). McLeod-Li tests for 2nd order nonlinear dependence.

#### 2.2.2 Tsay Test

Tsay (1986) proposed a test to detect the quadratic serial dependence in the data. Suppose *K =* k (k-1)/2 column vector contains all the possible cross products of the form r_t-1_ r_t-j_ where *ϵ* [i, k]. Thus,  and . Further, let  denote the projection of *v*_*t*,*i*_ on *r*_*t* - 1_ …, *r*_*t* - *k*_, on the subspace orthogonal to *r*_*t* - 1_, … *r*_*t* - *k*_ (the residuals from a regression of *v*_*t*,*i*_ on *r*_*t* - 1_, …, *r*_*t* - *k*_. Using following regression, the parameters *γ*_1_, … *γ*_*k*_ are estimated:
10

The Tsay F statistic is for testing the null hypothesis that *γ*_1_, … *γ*_*k*_ are all zero.

#### 2.2.3 ARCH-LM test

Engle (1982) proposed Lagrange Multiplier test to detect ARCH distributive. The test statistic based on R^2^ of an auxiliary regression, is defined as
11

When the sample size is *n*, under the null hypothesis of a linear generating mechanism for {e_t_}, the test statistic NR^2^ for this regression is asymptotically distributed, .

#### 2.2.4 Hinich bicorrelation test

The portmanteau bicorrelation test of Hinich (1996) is a third order extension of the standard correlation tests for white noise. The null hypothesis is that the transformed data {r_t_} are realizations of a stationary pure noise process that has zero bicorrelation (H). Thus, under the null, bicorrelations (H) are expected to be equal to zero. The alternative hypothesis is that the process has some non-zero bicorrelations (third order nonlinear dependence).
12

where . Z (t_k_) are standard observations at time t = k, and L = T^c^ with 0 < c < 0.5^e^.

#### 2.2.5 BDS test

Brock *et al.* (1996) developed a portmanteau test for time-based dependence in a series, popularly known as BDS (named after its authors). The BDS test uses the correlation dimension of Grassberger and Procaccia (1983). To perform the test for a sample of *n* observations {x_1_,..,x_n_}, an embedding dimension *m*, and a distance *ϵ*, the correlation integral C_m_ (n, ϵ) is estimated by
13

where *n* is sample size, *m* is embedding dimension and *ϵ* is the maximum difference between pairs of observations counted in estimating the correlation integral. The test statistic is:
14

The BDS considers the random variable √n(C_m_(n, ϵ) – C_1_(n, ϵ)^m^ which, for an *iid* process converges to the normal distribution as *n* increases. It has power against a variety of possible alternative specifications like nonlinear dependence and chaos. We use different *m*, and *ϵ* to estimate the BDS statistic.

## 3 Results and discussion

This section discusses the empirical results of both linear and nonlinear tests carried out in the present paper. Table 1 reports the descriptive statistics for Sensex and Nifty returns. The mean returns are positive during the full sample period and Sensex average returns were highest during 1991–93 while Nifty registered highest average returns in subsample 2003–05. The standard deviation of Nifty returns is greater than that of the Sensex. The former witnessed higher volatility during 2006–08 while the latter exhibited relatively higher volatility during 2000–2002 and 2006–08, the periods of financial and economic crises. The skewness is negative for the full sample and subsamples implying that returns are flatter to the left compared to the normal distribution. Moreover, it indicates that the negative returns have greater magnitude than the positive. The kurtosis indicates that return distribution has sharp peaks compared to a normal distribution. Further, Jarque and Bera (1980) statistic confirm that index returns are non- normally distributed.

**Table 1:** **Descriptive statistics**

Sample period	Mean	Minimum	Maximum	S.D	Skewness	Kurtosis	Jaqua-Bera
**Sensex**							
**Full sample**	0.000553	-0.136	0.159	0.017	-0.042	5.893	7780.03
Jan 1991 – Dec 1993	0.001988	-0.136	0.123	0.024	-0.047	4.624	541.93
Jan 1994 - Dec 1996	-0.000116	-0.046	0.056	0.014	0.454	1.229	68.16
Jan 1997 – Dec 1999	0.000656	-0.086	0.073	0.018	-0.086	2.091	135.45
Jan 2000 – Dec 2002	-0.000525	-0.074	0.071	0.017	-0.338	2.165	160.65
Jan 2003 – Dec 2005	0.001348	-0.118	0.079	0.013	-1.139	11.120	4075.26
Jan 2006 – Dec 2008	0.000035	-0.116	0.079	0.021	-0.344	2.584	222.00
Jan 2009 – Dec 2011	0.000635	-0.075	0.159	0.016	1.291	14.567	6766.81
Jan 2012- Mar 2013	0.000699	-0.027	0.026	0.009	0.077	0.580	5.014
**Nifty**							
**Full sample**	0.00035	-0.130	0.163	0.0162	-0.122	6.428	8262.46
Jan 1994 - Dec 1996	-0.0002	-0.043	0.054	0.0139	0.498	1.456	92.78
Jan 1997 – Dec 1999	0.0006	-0.088	0.099	0.0098	0.009	3.680	422.17
Jan 2000 – Dec 2002	-0.0004	-0.072	0.072	0.0160	-0.244	2.652	227.11
Jan 2003 – Dec 2005	0.0012	-0.130	0.079	0.0139	-1.407	12.870	5488.81
Jan 2006 – Dec 2008	0.0001	-0.130	0.067	0.0209	-0.530	3.298	372.82
Jan 2009 – Dec 2011	0.0006	-0.063	0.163	0.0156	1.403	15.912	8078.16
Jan 2012 – Mar 2013	0.0007	-0.027	0.027	0.0091	0.079	0.643	6.09

### 3.1 Linear dependence

The present study employs Ljung-Box test to check whether all autocorrelations are simultaneously equal to zero. The full samples of both the Sensex and Nifty possess autocorrelations indicating dependence in stock returns. The LB statistics are significant at 1 per cent level showing autocorrelations in the first two sub-periods. Nevertheless, stock returns in the last three subsamples follow a random walk. Interestingly, stock returns exhibit independence during 1997–1999 and 2000–2002, the years of Asian currency crisis and dot.com crash. The results for Nifty indicate first order autocorrelation in the first four subsamples except 1997–1999 and thus suggest the possibility of predictability of returns. Similar to that of Sensex, the results for Nifty during 2006–2008, 2009–2011 and 2012–2013 show no autocorrelations suggesting independence of returns. The runs tests statistics presented in the last column of Table 2 are significant at 1 per cent level and the negative values of Z for both Sensex and Nifty indicate positive correlation. The results show that during the first five subsamples, the null of the random walk is rejected with the exception in 1997–1999.

**Table 2:** **LB Q and runs tests statistics**

Sample periods	LB (5)	LB (15)	LB (20)	Runs Z Statistics
**Sensex**				
**Full sample**	-0.001	0.024	-0.023	-6.385*
	(46.45)*	(75.99)*	(96.84)*	
Jan 1991 – Dec 1993	0.086	0.113	0.055	- 3.528*
	(21.04)*	(52.22)*	(60.94)*	
Jan 1994 - Dec 1996	0.015	0.011	-0.052	- 4.236
	(38.39)*	(48.20)*	(50.89)*	
Jan 1997 – Dec 1999	-0.050	-0.020	-0.046	-1.842
	(3.73)	(17.04)	(22.41)	
Jan 2000 – Dec 2002	-0.022	0.006	-0.094	- 2.611*
	(6.34)	(14.42)	(31.77)**	
Jan 2003 – Dec 2005	-0.032	-0.056	0.010	- 2.358*
	(26.58)*	(35.45)*	(44.28)*	
Jan 2006 – Dec 2008	-0.017	0.011	-0.049	- 1.3356
	(7.60)	(15.23)		
Jan 2009 – Dec 2011	-0.055	0.002	-0.081	- 0.439
	(6.65)	(17.41)	(31.47)**	
Jan 2012 – Mar 2013	-0.008	0.009	0.024	- 0.929
	(2.15)	(12.78)	(22.54)	
**Nifty**				
**Full sample**	-0.008	0.001	-0.042	- 5.765*
	(34.69)*	(60.71)*	(91.60)*	
Jan 1994 - Dec 1996	0.030	0.003	-0.020	- 5.161*
	(44.35)*	(57.73)*	(59.53)*	
Jan 1997 – Dec 1999	0.002	-0.016	0.009	- 0.052
	(0.267)	(14.35)	(23.68)	
Jan 2000 – Dec 2002	0.016	0.013	-0.107	- 2.962*
	(12.74)**	21.02	38.90*	
Jan 2003 – Dec 2005	-0.037	-0.059	0.013	- 2.270**
	37.46*	55.30*	61.54*	
Jan 2006 – Dec 2008	-0.011	0.026	-0.066	- 1.105
	4.81	24.02	31.58**	
Jan 2009 – Dec 2011	-0.060	-0.006	-0.006	0.0367
	4.98	16.93	29.14	
Jan 2012 – Mar 2013	0.000	0.005	0.017	-0.874
	2.69	14.66	21.51	

The autocorrelation coefficient followed by Ljung-Box (LB) Q statistics in parenthesis are given in the table at lags 5, 15 and 20 for the full sample and subsample period. The null of LB is zero autocorrelation. The last column furnishes the Runs Z statistics. * and ** denote the significance level at 1% and 5% respectively.

The runs test results for the last three subsamples show no evidence of autocorrelation. We find no linear autocorrelations during those periods in which the major events namely, the East Asian financial crisis, dot.com bubble burst, and sub-prime mortgage crisis occurred. The autocorrelation and runs test results indicate that the Indian stock market is switching between efficiency and inefficiency. In other words, these results seem to support the view that Indian stock market is adaptive.

Furthermore, Lo and MacKinlay variance ratios at all the chosen investment horizons (*q*) for Sensex and Nifty during the full sample are greater than unity and statistically significant at 1 percent level, indicating returns do not follow a random walk (Table 3). Nevertheless, variance ratio statistics at any investment horizon in all the subsamples are insignificant indicating independence of returns in these sub-periods. The sequential procedure of Lo and MacKinlay (1988) test leads to size distortions and the test ignores the joint nature of random walk. To overcome this problem, The Chow-Denning test, statistically superior to individual variable ratio test, indicates predictability of stock returns in India by rejecting null of random walk over the whole sample at 5 per cent level of significance. However, every subsample provides evidence of the independence of returns. The individual and multiple variance ratio results suggest that the Indian market is largely efficient surrounded by brief periods of predictability which disappear because information quickly begins to reflect in returns and market moves towards efficiency again.

**Table 3:** **Variance ratio test statistics**

Sample periods	Lo-MacKinlay variance ratios for investment horizons (***q***)				Chow and Denning statistic
	2	4	8	16	
**Sensex**					
**Full sample**	1.08*	1.12*	1.12***	1.19**	3.767**
	(3.767)	(2.878)	(1.868)	(2.071)	
Jan 1991 – Dec 1993	1.11	1.20	1.26	1.42	1.066
	(1.066)	(1.083)	(0.910)	(1.001)	
Jan 1994 - Dec 1996	1.21	1.27	1.32	1.21	0.772
	(0.772)	(0.567)	(0.424)	(0.196)	
Jan 1997 – Dec 1999	1.04	1.08	1.03***	1.06	0.291
	(0.291)	(0.292)	(0.082)	(0.094)	
Jan 2000 – Dec 2002	1.06	1.09	1.10	1.11	0.391
	(0.391)	(0.298)	(0.211)	(0.163)	
Jan 2003 – Dec 2005	1.08	1.02	1.08	1.15	0.271
	(0.273)	(0.052)	(0.104)	(0.129)	
Jan 2006 – Dec 2008	1.07	1.07	0.985	1.05	0.653
	(0.653)	(0.346)	(-0.045)	(0.124)	
Jan 2009 – Dec 2011	1.06	1.06	1.01	1.09	0.319
	(0.319)	(0.172)	(0.022)	(0.117)	
Jan 2012 – Mar 2013	0.98	1.06	1.10	1.10	0.012
	(-0.01)	(0.023)	(0.024)	(0.018)	
**Nifty**					
**Full sample**	1.07*	1.08**	1.06	1.10	3.180*
	(3.180)	*(1.896)	(1.071)	(1.121)	
Jan 1994 - Dec 1996	1.23	1.31	1.40	1.25	1.055
	(1.055)	(0.789)	(0.673)	(0.304)	
Jan 1997 – Dec 1999	1.00	0.99	0.96	0.96	0.003
	(0.003)	(-0.005)	(-0.107)	(-0.068)	
Jan 2000 – Dec 2002	1.09	1.08	1.11	1.15	0.602
	(0.602)	(0.284)	(0.260)	(0.252)	
Jan 2003 – Dec 2005	1.11	1.06	1.12	1.16	0.587
	(0.586)	(0.183)	(0.218)	(0.203)	
Jan 2006 – Dec 2008	1.06	1.06	0.99	1.07	
	(0.677)	(0.395)	(-0.015)	(0.216)	0.677
Jan 2009 – Dec - 2011	1.04	1.05	1.00*	1.07	0.288
	(0.276)	(0.172)	(0.002)	(0.112)	
Jan 2012 – Mar 2013	0.97	1.06*	1.10**	1.12**	0.021
	(-0.021)	*(0.032)	(0.035)	(0.029)	

Note: The Lo-MacKinlay variance ratios VR (*q*) are reported in the main rows and variance test [Z * (*q*)] statistics are given in parentheses. Under the null of random walk, the variance ratio value is expected to equal one. Chow-Denning heteroscedastic statistics are presented in the last column and the critical value is 2.49. *, ** and *** denote significance at 1%, 5% and 10% respectively.

**Figure 1:**
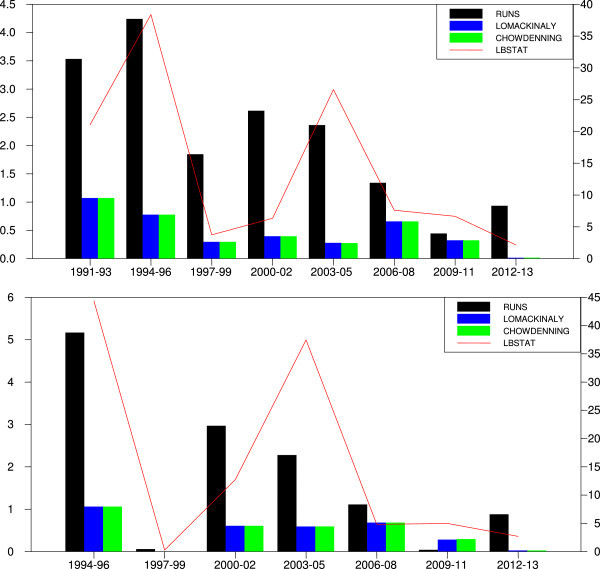
**Trends in linear tests statistics.**

The trends in linear test statistics help to examine the magnitude of linear dependence during the sample period (Figure 1). For Sensex, LB statistics witness sharp upward and downward spikes during the sample period. The test statistics were highest during 1994–1996 and 2003–2005. Notwithstanding, the LB Q statistics started moving downward from 2006 including the periods of sub-prime mortgage crisis and global economic meltdown of 2008. The trends in runs statistics exhibit similar patterns. The Lo-MacKinlay and Chow-Denning statistics show that the magnitude of linear dependence is highest during the first two subsamples, 1994–1996, 1997–1999. Thereafter, the trend in test statistics moving downwards, and values are insignificant indicating no predictability of returns based on past returns. The trends in magnitude of linear dependence in Nifty returns are similar to that of Sensex. The linear test results presented in Figure 1 indicate highest linear dependence in Nifty returns during subsample 1994–1996 and 2003–2006. In the rest of the subsamples, the values are low showing no autocorrelation or linear dependence in Nifty returns. Overall, the magnitude of linear dependence has fallen over the period (Figure 1). In other words, the results support that the Indian stock market has become efficient from the beginning of the year 2003. It may be because of the fact that NSE has brought several changes in market microstructure and trading practices, which BSE followed later. It seems that these changes along with financial sector reforms and regulatory measure of Securities and Exchange Board of India (SEBI) have positively influenced the efficiency in the market. Strikingly, linear test statistics are statistically insignificant during 1997–1999, 2000–02 and 2006–2008, the periods of Asian financial crash, tech boom burst and sub-prime mortgage crisis followed by a global recession respectively. The present evidence of unpredictability of returns during crises is consistent with Kim *et al*. (2011) who observed no predictability during stock market crashes (1929 and 1987).

### 3.2 Nonlinearity in stock returns

The linear tests such as autocorrelation, variance ratio, and runs tests are incapable to capture nonlinear patterns in the return series. The failure to reject linear dependence is insufficient to prove independence in view of non-normality of the series (Hsieh 1989) and not necessarily imply independence (Granger and Anderson 1978). The presence of nonlinearity indicates predictability and potential excess profits to agents. The use of linear models in such conditions may give the wrong inference of unpredictability. Moreover, the presence of nonlinearity in stock returns contradicts EMH. In this study, we employed a set of nonlinear tests to investigate the presence of nonlinear dependence. Before performing these tests, the returns were corrected for heteroscadasticity and we removed linear dependence fitting an appropriate AR (*ρ*) model so that any remaining dependence would be nonlinear. LB statistics for residuals extracted after filtering for linearity show no autocorrelation up to lag 20 for each subsample of Sensex and Nifty (Table 4).

**Table 4:** **McLeod-Li test statistics**

Sample periods	AR (***ρ***)	LB (5)	LB (15)	LB (20)	McLeod-Li statistic		
					Lag 5	Lag 15	Lag 20
**Sensex**							
**Full sample**	9	0.043	0.748	26.32	988.6*	2130.1*	2415.5*
		(1.000)	(1.000)	(0.155)	(0.000)	(0.000)	(0.000)
Jan 1991 – Dec 1993	7	0.196	24.06	29.57	81.7*	238.0*	255.3*
		(0.999)	(0.064)	(0.077)	(0.000)	(0.000)	(0.000)
Jan 1994 - Dec 1996	2	4.745	16.94	19.14	47.17*	97.49*	130.53*
		(0.447)	(0.322)	(0.512)	(0.000)	(0.000)	(0.000)
Jan 1997 – Dec 1999	1	3.64	16.86	22.38	30.19*	41.84*	52.99*
		(0.602)	(0.327)	(0.320)	(0.000)	(0.000)	(0.000)
Jan 2000 – Dec 2002	2	3.161	11.00	26.48	187.69*	296.07*	329.56*
		(0.675)	(0.752)	(0.150)	(0.000)	(0.000)	(0.000)
Jan 2003 – Dec 2005	2	8.673	20.459	23.306	245.29*	263.26*	264.19*
		(0.122)	(0.155)	(0.274)	(0.000)	(0.000)	(0.000)
Jan 2006 – Dec 2008	2	2.349	18.209	23.168	277.27*	590.73*	671.05*
		(0.798)	(0.251)	(0.280)	(0.000)	(0.000)	(0.000)
Jan 2009 – Dec - 2011	1	6.263	18.81	23.67	4.712	29.28**	33.42**
		(0.281)	(0.222)	(0.296)	(0.451)	(0.014)	(0.030)
Jan 2012 – Mar 2013	0	2.152	12.788	22.546	1.550	15.00	27.79
		(0.827)	(0.618)	(0.311)	(0.907)	(0.451)	(0.114)
**Nifty**							
**Full sample**	11	0.028	6.371	26.939	550.20*	964.60*	1066.23*
		(1.000)	(0.972)	(0.137)	(0.000)	(0.000)	(0.000)
Jan 1994 - Dec 1996	2	5.813	19.919	21.824	69.38*	154.97*	185.82*
		(0.324)	(0.175)	(0.350)	(0.000)	(0.000)	(0.000)
Jan 1997 – Dec 1999	0	0.267	14.356	23.686	23.72*	28.13**	49.47*
		(0.998)	(0.498)	(0.256)	(0.000)	(0.020)	(0.000)
Jan 2000 – Dec 2002	2	1.429	7.764	22.700	108.87*	199.44*	220.53*
		(0.921)	(0.932)	(0.303)	(0.000)	(0.000)	(0.000)
Jan 2003 – Dec 2005	2	8.715	21.42	28.444	286.24*	310.20*	311.07*
		(0.157)	(0.321)	(0.099)	(0.000)	(0.000)	(0.000)
Jan 2006 – Dec 2008	2	1.593	19.37	26.913	232.57*	441.67*	489.24*
		(0.902)	(0.197)	(0.137)	(0.000)	(0.000)	(0.000)
Jan 2009 – Dec - 2011	2	3.379	14.305	26.676	2.177	18.701	22.491
		(0.641)	(0.502)	(0.144)	(0.824)	(0.227)	(0.314)
Jan 2012 – Mar 2013	0	2.697	14.663	21.516	2.667	16.101	30.169***
		(0.746)	(0.475)	(0.367)	(0.751)	(0.375)	(0.067)

The autocorrelation coefficient followed by The Ljung-Box (LB) Q statistics in parenthesis are given in the table at lags 5, 15 and 20 for the full sample and subsample period. *, ** and *** denote significance at 1%, 5% and 10% respectively.

The McLoed-Li statistics prove that each subsample of Sensex and Nifty has a nonlinear dependence except 2012–13 and 2009–2013 subsamples. This indicates that Indian stock market is inefficient during these sample periods and over the whole sample period. Further, Tsay and Engle LM tests show strong evidence of nonlinear behaviour for both the full sample and subsamples at chosen lags (Table 5). Similar to McLeod-Li results, the Tsay and Engle LM tests indicate unpredictability of returns during the last subsample (2012–13). Overall, the results presented in Tables 4 and 5 show a significant presence of nonlinearity in returns. This implies that Indian stock market was weakly inefficient throughout the sample period.

**Table 5:** **Tsay, Engle LM and H statistics**

Sample period	AR (***ρ***)	Tsay F statistic			Engle LM statistic			H statistic
		Lag 5	Lag 15	Lag 20	Lag 5	Lag 15	Lag 20	
**Sensex**								
**Full sample**	9	7.862*	3.613*	3.039*	564.1*	729.5*	758.2*	3760.9*
		(0.000)	(0.000)	(0.000)	(0.000)	(0.000)	(0.000)	(0.000)
Jan 1991 – Dec 1993	7	2.837*	1.907*	1.786*	54.8*	101.2*	110.5*	405.6*
		(0.000)	(0.000)	(0.000)	(0.000)	(0.000)	(0.000)	(0.000)
Jan 1994 - Dec 1996	2	1.858*	1.273**	1.282**	32.4*	50.5*	74.9*	139.7*
		(0.000)	(0.041)	(0.016)	(0.000)	(0.000)	(0.000)	(0.000)
Jan 1997 – Dec 1999	1	2.436*	1.686*	1.457*	28.86*	37.5*	47.7*	183.9*
		(0.001)	(0.000)	(0.000)	(0.000)	(0.001)	(0.005)	(0.000)
Jan 2000 – Dec 2002	2	2.396*	2.433*	2.168*	110.67*	138.8*	148.9*	364.8*
		(0.002)	(0.000)	(0.000)	(0.000)	(0.000)	(0.000)	(0.000)
Jan 2003 – Dec 2005	2	6.609*	2.257*	1.910*	268.96*	272.2*	272.3*	721.7*
		(0.000)	(0.000)	(0.000)	(0.000)	(0.000)	(0.000)	(0.000)
Jan 2006 – Dec 2008	2	4.734*	2.746*	2.667*	153.7*	179.4*	181.7*	680.9*
		(0.000)	(0.000)	(0.000)	(0.000)	(0.000)	(0.000)	(0.000)
Jan 2009 – Dec - 2011	1	1.24	2.50*	2.483*	4.9	22.8	24.3	242.9*
		(0.229)	(0.000)	(0.000)	(0.495)	(0.088)	(0.231)	(0.000)
Jan 2012 – Mar 2013	0	0.560	1.558*	1.073	1.6	13.3	25.8	52.8
		(0.903)	(0.003)	(0.359)	(0.911)	(0.576)	(0.172)	(0.198)
**Nifty**								
**Full sample**	9	6.240*	2.877*	2.427*	352.20*	425.36	437.38*	1848.41*
		(0.000)	(0.000)	(0.000)	(0.000)	(0.000)	(0.000)	(0.000)
Jan 1994 - Dec 1996	2	1.509	1.583*	1.436*	50.21*	77.758	92.62*	158.67*
		(0.095)	(0.000)	(0.000)	(0.000)	(0.000)	(0.000)	(0.000)
Jan 1997 – Dec 1999	0	2.842*	1.687*	1.295**	24.21*	27.85**	51.658*	157.77*
		(0.000)	(0.000)	(0.011)	(0.000)	(0.022)	(0.000)	(0.000)
Jan 2000 – Dec 2002	2	1.852**	2.173*	1.949*	79.97*	126.46*	130.54*	380.80*
		(0.024)	(0.000)	(0.000)	(0.000)	(0.000)	(0.000)	(0.000)
Jan 2003 – Dec 2005	2	6.757*	2.413*	1.985*	315.46*	320.34*	321.86*	799.69*
		(0.000)	(0.000)	(0.000)	(0.000)	(0.000)	(0.000)	(0.000)
Jan 2006 – Dec 2008	2	5.583*	2.705*	2.459*	125.40*	152.41*	158.98*	663.08*
		(0.000)	(0.000)	(0.000)	(0.000)	(0.000)	(0.000)	(0.000)
Jan 2009 – Dec - 2011	2	0.873	2.313*	2.268*	2.023	15.292	16.87	195.63*
		(0.593)	(0.000)	(0.000)	(0.845)	(0.430)	(0.661)	(0.000)
Jan 2012 – Mar 2013	0	0.489	1.577*	1.181	2.931	14.672	27.786	56.255
		(0.945)	(0.003)	(0.193)	(0.710)	(0.475)	(0.114)	(0.121)

*,** denote 1% and 5% significance level.

The H statistics reject null of pure noise for Sensex and Nifty in all the subsamples with the exception of subsample 2012–2013 (Table 5). This exposes nonlinear characteristics of Indian stock returns during these periods. Finally, the BDS statistics support evidence of nonlinear dependence during the subsamples and full sample for both the indices (Table 6). The rejection for residuals from AR (*ρ*) indicates presence of nonlinear dependence and implies the possible predictability of future returns using the history of returns.

**Figure 2:**
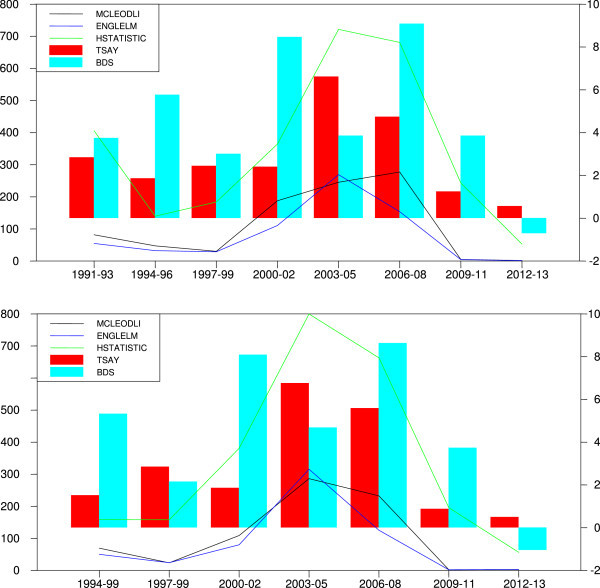
**Trends in nonlinear tests statistics.**

The trends in McLeod-Li show stronger presence of nonlinear dependence in Sensex and Nifty returns during subsamples 2003–2005 and 2006–2008 (Figure 2). Again, the trends in Engle LM, Tsay, H and BDS test statistics are low indicating lesser magnitude of nonlinear dependency in stock returns till the year 2000 and thereafter returns exhibit increasing nonlinear tendency reaching peak during subsample, 2006–2008. The subsample 2006–2008 that possess pockets of strong presence of nonlinear dependence is associated with sub-prime mortgage and global financial crisis (2008). In post 2008 subsample, however, all the test statistics suggest relatively weaker presence of nonlinear dependence in returns (See, Figure 2).

**Figure 3:**
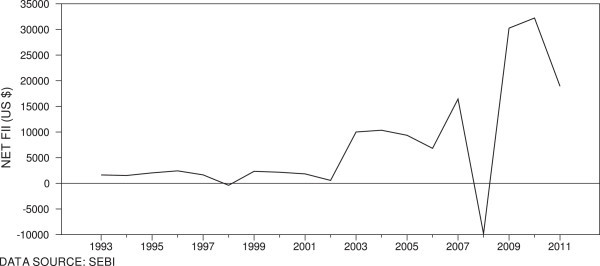
**Foreign institutional investments in Indian stock market.**

The foreign portfolio investments help emerging markets by offering quality information and liquidity and influence efficiency. Nevertheless, the yield sensitive FIIs fly from emerging markets in response to global news or loss of confidence in the economy. We find interesting association between nonlinear dependence and FIIs in India. We find net outflow of FIIs from Indian stock market creating panic in the market during financial crises of 1997–98, 2000–01 and 2007–08 and during these periods, we find statically significant nonlinearity in stock returns (Figure 3). The external events thus influence the behaviour of returns in emerging markets. Overall, we find a strong evidence of nonlinearity throughout the sample period in Indian market. Although we find evidence of an increasing nonlinear dependence, it is tapering off in most recent subsamples.

**Table 6:** **BDS test statistics**

Sample period	AR (***ρ***)	m = 2, ϵ = 0.75s	m = 4, ϵ = 1.0s	m = 8, ϵ =1.25***S***	m = 10, ϵ = 1.50s
**Sensex**					
**Full sample**	9	17.65* (0.000)	27.88* (0.000)	42.94*(0.000)	44.24* (0.000)
Jan 1991 – Dec 1993	7	3.74* (0.000)	4.42*(0.000)	6.33*(0.000)	7.40*(0.000)
Jan 1994 - Dec 1996	2	5.76* (0.000)	8.24* (0.000)	11.66* (0.000)	12.21*(0.000)
Jan 1997 – Dec 1999	1	3.00* (0.002)	4.52* (0.000)	6.30*(0.000)	6.95* (0.000)
Jan 2000 – Dec 2002	2	8.46*(0.000)	13.08*(0.000)	18.11*(0.000)	19.20*(0.000)
Jan 2003 – Dec 2005	2	3.85* (0.000)	5.60* (0.000)	9.01*(0.000)	9.77* (0.000)
Jan 2006 – Dec 2008	2	9.08* (0.000)	14.26*(0.000)	24.40*(0.000)	23.56*(0.000)
Jan 2009 – Dec - 2011	1	3.85* (0.000)	7.40*(0.000)	12.98* (0.000)	14.11* (0.000)
Jan 2012 – Mar 2013	0	-0.71* (0.474)	0.306* (0.759)	1.893* (0.058)	2.288* (0.022)
**Nifty**					
**Full sample**	11	15.15* (0.000)	23.89 (0.000)	35.94 (0.000)	37.65 (0.000)
Jan 1994 - Dec 1996	2	5.322* (0.000)	8.534* (0.000)	11.33* (0.000)	11.73* (0.000)
Jan 1997 – Dec 1999	0	2.149* (0.031)	4.081* (0.000)	5.351* (0.000)	5.808* (0.000)
Jan 2000 – Dec 2002	2	8.08* (0.000)	12.14* (0.000)	15.28* (0.000)	15.59* (0.000)
Jan 2003 – Dec 2005	2	4.67* (0.000)	6.43* (0.000)	9.68* (0.000)	10.89* (0.000)
Jan 2006 – Dec 2008	2	8.63* (0.000)	13.89* (0.000)	23.71* (0.000)	22.49* (0.000)
Jan 2009 – Dec - 2011	2	3.73* (0.000)	6.61* (0.000)	12.02* (0.000)	12.97* (0.000)
Jan 2012 – Mar 2013	0	-1.05 (0.292)	0.288 (0.772)	1.732 (0.083)	2.905 (0.004)

Here, ‘*m*’ and ‘*ϵ*’ denote the embedding dimension and distance, respectively and ‘ϵ’ equal to various multiples (0.75, 1, 1.25 and 1.5) of standard deviation (*scp* of the data. The value in the first row of each cell is a BDS test statistic followed by the corresponding p-value in parentheses. The asymptotic null distribution of test statistics is N (0.1). Asterisked values indicate 1% level of significance.

The linear test results support the proposition that Indian stock market switched between efficient and inefficient periods and this finding is consistent with Charles et al. (2012) Kim *et al.* (2011), and Alvarez-Ramirez *et al.* (2012). Nevertheless, the present evidence of strong presence of nonlinear dependence in stock returns throughout the sample suggests that the Indian stock market still inefficient and not experienced efficiency yet. Our finding of highest magnitude of nonlinearity during periods of financial crises in Indian stock returns is consistent with the findings of Urquhart and Hudson (2013) who found similar evidence for the US market. The evidence of nonlinearity during financial crises shows that the stock market crash and economic crises negatively affect the stock market efficiency. Furthermore, the present study finds outflow of FIIs during global financial crises. This evidence suggests that the increasing integration of Indian capital market has not only brought the benefits but also exposed the market to external shocks.

## 4 Summary and conclusion

The present paper has investigated the adaptive market hypothesis (AMH) in India, one of the fastest growing markets. The linear test results indicated a cyclical pattern in autocorrelations suggesting that the Indian stock market switched between periods of efficiency and inefficiency and market has become efficient from the year 2003. The findings from each of the nonlinear tests suggest a strong presence of nonlinear dependence in Indian stock returns throughout the sample period implying possible predictability of returns and consequent excess returns. The nonlinearity in stock returns was highest during various financial crises originated outside India and this finding shows association of informational inefficiency and financial crises. Furthermore, the vulnerability of Indian stock market to the external shocks in a financially liberalized economy is evident from the outflow of FIIs owing to external events. The present evidence of influence of financial crises and reversal of FIIs on efficiency of stock market should be interpreted as identifying an association rather than causality.

The reforms initiated have positive influence on stock market is evident from the fall in magnitude of nonlinear dependence in recent periods. The present study finds that Indian stock market is still evolving and not fully adaptive, as it has not gone through a single period of efficiency. The linear independence and weaker presence of nonlinear dependence in returns from 2009 is sufficient to conclude that Indian stock market is moving towards efficiency. The present finding of an increased possibility of predictability during financial crises and large outflow of investment calls for appropriate policy measures to address the external shocks and retain the confidence of foreign investors.

### Endnotes

^a^The seminal work of Bachelier (1900) laid theoretical foundation for the theory of efficient market. The pioneering work of Samuelson (1965) added rigour to the theory of stock market efficiency.

^b^Malkiel (1973) writes to the extent that ‘a blindfolded chimpanzee throwing darts at the Wall Street could select a portfolio that would do as well as the experts’.

^c^Campbell *et al.* (1997) note that testing of market efficiency as a condition of all or nothing is not useful and such an efficient market is the economically unrealizable ideal market. They suggest relative efficiency because measuring efficiency provides more insights than testing it.

^d^A rolling sample is an alternative method used in empirical literature to examine evolving nature. However, an event may squeeze or influence the overall results. Hinich and Patterson (1995) suggested a windowed approach. We did not find any optimization benefits in using rolling sample in the present context.

^e^Hinich and Patterson in their unpublished work of 1995 recommend c = 0.4. The same is followed here.
